# Machine learning methodology for high throughput personalized neutron dose reconstruction in mixed neutron + photon exposures

**DOI:** 10.1038/s41598-021-83575-5

**Published:** 2021-02-17

**Authors:** Igor Shuryak, Helen C. Turner, Monica Pujol-Canadell, Jay R. Perrier, Guy Garty, David J. Brenner

**Affiliations:** Center for Radiological Research, Columbia University Irving Medical Center, 630 West 168th street, VC-11-234/5, New York, NY 10032 USA

**Keywords:** Computational biology and bioinformatics, Machine learning, Cell biology

## Abstract

We implemented machine learning in the radiation biodosimetry field to quantitatively reconstruct neutron doses in mixed neutron + photon exposures, which are expected in improvised nuclear device detonations. Such individualized reconstructions are crucial for triage and treatment because neutrons are more biologically damaging than photons. We used a high-throughput micronucleus assay with automated scanning/imaging on lymphocytes from human blood ex-vivo irradiated with 44 different combinations of 0–4 Gy neutrons and 0–15 Gy photons (542 blood samples), which include reanalysis of past experiments. We developed several metrics that describe micronuclei/cell probability distributions in binucleated cells, and used them as predictors in random forest (RF) and XGboost machine learning analyses to reconstruct the neutron dose in each sample. The probability of “overfitting” was minimized by training both algorithms with repeated cross-validation on a randomly-selected subset of the data, and measuring performance on the rest. RF achieved the best performance. Mean R^2^ for actual *vs.* reconstructed neutron doses over 300 random training/testing splits was 0.869 (range 0.761 to 0.919) and root mean squared error was 0.239 (0.195 to 0.351) Gy. These results demonstrate the promising potential of machine learning to reconstruct the neutron dose component in clinically-relevant complex radiation exposure scenarios.

## Introduction

Implementation of rapidly-evolving machine learning techniques provides opportunities for improvements in multiple disciplines^1–3^, particularly where the amount of data to be analyzed is large and variables interact with each other in complex nonlinear ways. The field of radiation biodosimetry is a good candidate for benefitting from machine learning applications, because its goal is to rapidly produce individualized reconstructions of the radiation dose and biological damage magnitude based on samples (e.g. blood) obtained from large numbers of people affected by a large-scale radiological event such as an improvised nuclear device (IND) detonation^4–13^. Increasing recognition of the potential of machine learning in radiation biodosimetry is reflected in a growing number of publications^14–17^.

One of the most challenging problems in radiation biodosimetry is posed by exposures to mixtures of densely ionizing neutrons and sparsely ionizing photons (e.g. gamma rays) after IND detonation. The radiation quality and type of exposure can vary between individuals, and neutrons can account for up to ~ 30% of the total dose^4,18^. Quantitative reconstruction of the neutron component in such mixed exposures is very important for accurate triage, treatment and prognosis of the affected individuals, because neutrons are much more biologically damaging per unit dose than photons^19^. Neutron relative biological effectiveness (RBE) tends to be particularly high for some delayed consequences of irradiation, such as carcinogenesis^20–22^. Consequently, the neutron component of a mixed exposure can potentially cause a considerable portion or even the majority of deleterious radiation effects.

Cytogenetic methods—dicentric chromosome (DCA) and cytokinesis-block micronucleus (CBMN) assays—represent established and robust radiation biodosimetry tools because of low background yields in unirradiated individuals and reliable radiation dose responses. Development of these techniques continues throughout the world^23–25^. High-throughput automated approaches using scanning and imaging software are available to implement these assays^26–28^.

Traditional biodosimetry approaches based on mean yields of cytogenetic damage per cell are good at estimating total absorbed dose, but the mean does not provide sufficient information to discriminate between neutron and photon components of a mixed exposure. Our approach in this study involves combining the CBMN assay with machine learning techniques such as random forests (RF) to quantitatively reconstruct the neutron dose in mixed neutron + photon exposures, based on the probability distribution of cytogenetic damage in ex vivo irradiated peripheral blood lymphocytes.

Previously we showed that these probability distributions for micronuclei (MN) per binucleated cell differ between photon and neutron exposures, and performed proof of principle calculations for several endpoints (e.g. whole-body vs partial-body photon exposure, presence of neutrons in the total dose) using this information^17^. Good performance was achieved for classifying mixed exposures by presence or absence of neutrons in a binary sense (i.e. neutron contributions of < 10% or ≥ 10% of the total dose), but performance was weaker for the task of quantitative reconstruction of the neutron dose component in mixed neutron + photon exposures. The objective of the present work was to improve our solution to the latter problem by focusing on quantitative neutron dose reconstruction in mixed exposures as the main task, treating neutron dose as a continuous variable and refining the machine learning analysis specifically for this purpose.

**Figure 1:**
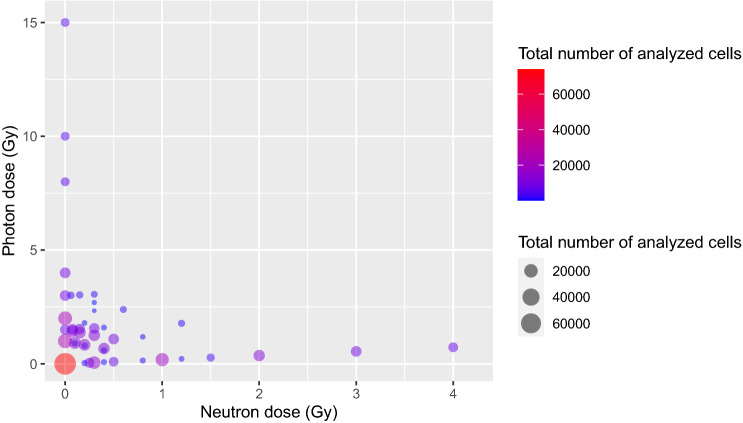
Summary of the analyzed data set on radiation-induced micronuclei in ex vivo irradiated peripheral blood lymphocytes. Each circle represents a specific combination of neutron and/or photon doses. The same data are listed numerically in Supplementary Table S1.

To meet this objective, we expanded our micronuclei data set (summarized in Supplementary Table S1, Fig. 1) to test notably larger numbers of blood samples and different combinations of neutrons with photons (44 combinations, 542 blood samples). This expanded version includes higher doses of neutrons and photons, whereby the maximum neutron dose was increased from 3 to 4 Gy, and the maximum photon dose was increased from 4 to 15 Gy, compared with our previously published data^17^. Importantly, the data set now includes high doses where the decreased cell proliferation results in lower than expected micronucleus yield, with mean values starting to decrease with dose^23^. The enhanced data set allows to test the ability of our proposed methodology to distinguish high photon doses from neutron exposures. Based on the results, which are presented below, we argue that such approaches can achieve high accuracy of neutron component reconstruction in mixed neutron + photon exposures and are promising for high throughput radiation biodosimetry.

## Materials and methods

### Data set

The methodologies of blood sample collection and neutron and photon irradiation were described in detail in our previous publications^17,23^. Briefly, peripheral blood samples were collected by venipuncture into 6 ml lithium-heparinized Vacutainer tubes (BD Vacutainer, Franklin Lakes, NJ) from healthy female and male donors (non-smokers with no known exposure to X rays or CT scans within the last 12 months), with informed consent as approved by the Columbia University Medical Center Institutional Review Board (IRB protocols AAAE-2671 and AAAR-9996). All methods were performed in accordance with the relevant guidelines and regulations. Neutron irradiation of these blood samples was performed at the Columbia IND Neutron Facility (CINF) using an IND-mimicking neutron energy spectrum^29–31^. Dose rates for neutron irradiations varied between 1.3 and 2.6 Gy/h. The gamma ray dose component in these exposures was approximately 18%.

For mixed neutron + photon irradiations with lower fractions of neutrons, blood samples were exposed to 1.23 Gy/min of 250-kVp X-rays (15 mA; 1.2 Gy/min; 0.5 mm Cu, 1 mm Al; HVL = 2 mm Cu) from a Westinghouse Coronado orthovoltage X-ray irradiator, 5–10 min following neutron irradiation. For pure photon irradiation, samples of whole blood were either irradiated using the same Coronado machine or using a Gammacell 40 ^137^Cs irradiator (Atomic Energy of Canada, Ltd., Chalk River, Canada) at a dose rate of 0.73 Gy/min. Dose rate for the Coronado was verified on the day of exposure, using a Victoreen R chamber. The dose rate for the Gammacell is verified annually using thermoluminescent dosimeters (TLDs). The homogeneity of the exposure across the sample volume, in both cases was verified using EBT3 Gafchromic film (Ashland Advanced Materials, Bridgewater, NJ), which indicated less than 5% variation within the sample.

The CBMN assay protocol, which uses small volumes of blood in multi-well plate format, and subsequent imaging analysis and micronuclei scoring, are described in our earlier publications^17,28,30^. The resulting data set assembled for the current study was substantially expanded since our previous analysis^17^. It is summarized in Supplementary Tables S1, S2 and Fig. 1, and provided in full in the Supplementary_Dataset_File Supplementary_Dataset_File. Unfortunately, combinations of high neutron doses with high photon doses could not be evaluated because they resulted in very severe damage to the cells, causing the majority of the cells to never reach the binucleated state, where micronuclei can be scored. However, we were able to include separate high neutron doses (up to 4 Gy) and high photon doses (up to 15 Gy) in the data set. The mean number of analyzed cells per neutron + photon dose combination was 8423 (range 193 to 73,849), and the mean number of cells per sample was 684 (range 33 to 3561). The MN/cell yields varied from 0.062 to 0.535.

### Machine learning approach

We manually engineered 27 “features” from the raw data (Supplementary_Dataset_File) based on our judgement of what metrics could act as reasonable potential predictors of neutron dose in mixed neutron + photon exposure scenarios. These predictors, described in Table 1, were motivated by the available published literature about overdispersion (relative to the Poisson distribution) of cytogenetic damage probability distributions induced by densely ionizing radiations^32,33^.

**Table 1:** Descriptions of predictor variables used in our analyses for neutron dose reconstruction.

Name	Definition
Frac_0, … Frac_5	Fractions of cells with indicated number of MN, from 0 to 5
FracSq_0, … FracSq_5	Frac_0^2^ to Frac_5^2^, respectively
LnSum	Ln of the sum of analyzed cells per sample
LnMean	Ln of the mean number of micronuclei per cell
LnVar	Ln of the variance of the number of micronuclei per cell
LnVar_p	(exp[LnVar])^6^ = Var^6^
LnVarMean	Ln of the variance divided by the mean, = LnVar—LnMean
LnVarMean_p	10 × LnVarMean^6^
LnZeroFrac	ln[1 + Frac_0], where Frac_0 is the fraction of cells with 0 micronuclei
Ln3Frac	ln[1 + f_3_], where f_3_ is the fraction of cells with ≥ 3 micronuclei
Ln3Frac_p	10^6^ × Ln3Frac^6^
LnFD	Fisher dispersion index, calculated according to the following equation ^41^, where *M* is the sample mean, V is the variance, and n is the sample size: $LnFD = {\text{ln}}\left[ {\left( {\frac{1}{{\sqrt {2n} }}\left[ {\left( {n - 1} \right)\frac{V}{M} - n} \right]} \right)^{2} } \right]$
LnFD_neg	-LnFD
SEK	Sample excess kurtosis, calculated using the following equation, where z_i_ are standardized data values using the standard deviation based on the sample size *n* rather than on *n* − 1: $SEK = {\frac{1}{n}\mathop \sum \limits_{i = 1}^{n} z_{i}^{4} - 3}$
LnSkew	Sample skewness, defined as LnSkew = ln[m_3_/SD^3^], where m_3_ is the sample third central moment and SD is its standard deviation
LL_exp_Pois_dif	The difference in maximized log likelihoods for fitting an Exponential distribution to the sample data *vs.* the Poisson distribution, calculated as follows, where *k* is the MN count value in the *i*-th cell and *M* is the sample mean: $LLexp = \mathop \sum \limits_{i = 1}^{n} - \left( {k + 1} \right)\ln \left[ {1 + M} \right] + k {\text{ln}}\left[ M \right]$ $LLPois = \mathop \sum \limits_{i = 1}^{n} k\ln \left[ M \right] - M - {\text{ln}}\left[ {k!} \right]$ ${\text{LL}}\_{\text{exp}}\_{\text{Pois}}\_{\text{dif }} = \left( {{\text{LLexp}} - {\text{LLPois}}} \right)/{\text{n}}$
LL_exp_Pois_dif_p	10^6^ × LL_exp_Pois_dif^6^

The prefix “Ln” indicates natural logarithm. M is the mean, V is the variance, and n is the number of cells in the analyzed sample. The predictor variables were selected based on our judgement, combined with information about overdispersion of cytogenetic damage from densely ionizing radiation exposures^32,33^. Some of the predictors represent versions of the same concept (e.g. LnVar and LnVar_p, LnFD and LnFD_neg). They were used because the random forest algorithm (described in the main text) can use only a subset of predictors in different decision trees to reduce correlations between predictions of different trees, so that only one version of a given predictor (not both) could be present in some trees.

For example, the Poisson and Exponential distributions are two simple one-parameter distributions (where the mean is the only parameter) with different shapes. The Poisson distribution (*P*_*Pois*_), and a modification of the Exponential distribution for discrete variables (*P*_*Exp*_), are described by the following equations, where *k* is the number of MN/cell and *M* is the mean:

1 $$P_{Pois} = \frac{{M^{k} \times \exp \left[ { - M} \right]}}{k!}, P_{Exp} = \frac{{\left( {1 + \frac{1}{M}} \right)^{ - k} }}{1 + M}.$$

**Figure 2:**
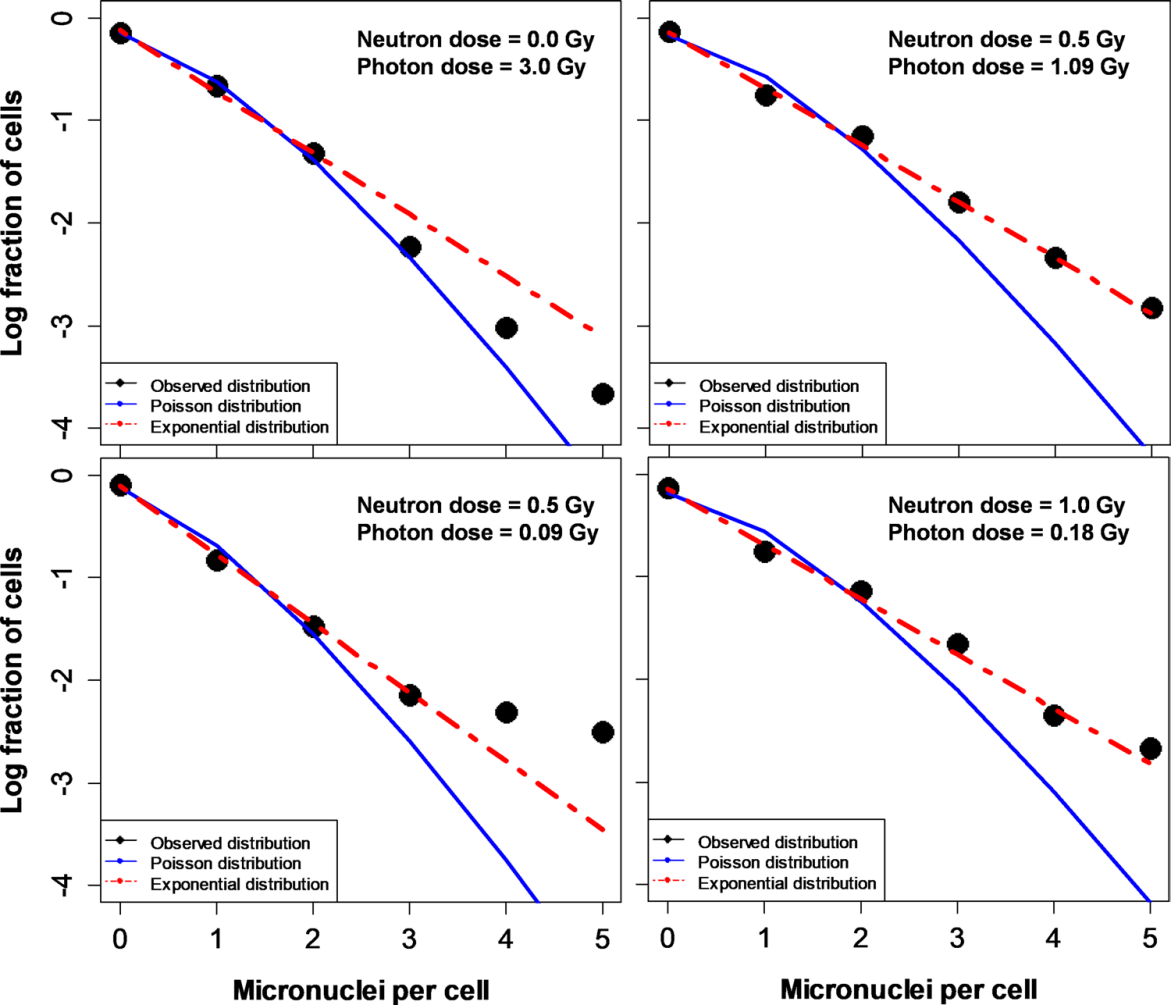
Comparison of some real data examples from our data set with the Poisson and Exponential distributions. These examples show the tendency of the data to deviate away from the Poisson distribution and to approach an Exponential distribution in blood samples exposed to substantial neutron doses. In contrast, data from samples exposed to photons only tend to be closer to the Poisson distribution.

Inspection of various real data examples from our data set suggests that the observed MN/cell probability distribution tended to be much closer to Exponential than to Poisson in those blood samples that were irradiated with substantial neutron doses, whereas pure photon exposures resulted in distributions closer to Poisson (Fig. 2). These differences in distribution shapes based on the magnitude of the neutron contribution are apparent even though the mean MN/cell values in these examples were relatively similar: between 0.27 and 0.41. To quantify this phenomenon and use it as a predictor for machine learning-based neutron dose reconstruction, for each blood sample we compared whether the observed distribution is closer to Poisson or Exponential using maximum likelihood. This approach resulted in the predictor variable called LL_exp_Pois_dif, which is described in Table 1.

The 27 engineered predictors (listed in Table 1) were calculated for each blood sample, and the resulting data set was randomly split into training and testing halves using the *caret* package in *R* 4.0.2 software^34^. On the training data, we selected which predictors to keep for further analysis, and which to discard. The selection procedure consisted of manual and automatic steps. In the first manual step, we constructed a correlation matrix of all variables in the data set based on Spearman’s correlation coefficients, calculated the p-value for each coefficient, and visualized the results. We discarded those predictor variables that had weak correlations with neutron dose and/or strong correlations with each other.

After dropping these weak and redundant predictors, the remaining predictor set was analyzed by the Boruta feature selection algorithm to generate a ranking of importance scores for the predictor variables. Boruta iteratively compares the importance score of each predictor with the importance score of its randomly shuffled “shadow”^35^. It duplicates the data set, and shuffles the values in each column. These shuffled values are called shadow features, which are re-created in each iteration. Those predictors that have significantly (p-value < 0.01) worse importance than shadow ones are consecutively dropped. The Boruta analysis was repeated 100 times with different initial random number seeds, and the predictor variables were ranked by the median of median importance scores across all repeats.

Those predictor variables that passed the manual and Boruta-based screening were used for neutron dose reconstructions. As an initial test of whether or not the selected predictor set is reasonable, we performed a robust linear regression on the training data, with neutron dose as the dependent variable and the selected predictors as independent variables. Performance of this regression was measured on the testing data by calculating the coefficient of determination (R^2^) and root mean squared error (RMSE) between actual and reconstructed neutron doses. The regression showed decent performance (R^2^ = 0.77, RMSE = 0.32 Gy), suggesting that more flexible machine learning methods that can model complex non-linear relationships and interactions between predictors could achieve even better results on these data.

We selected two powerful and commonly-used machine learning methods: random forest (RF)^36^ and extreme gradient boosting (XGboost)^37^. Both are ensemble methods which fit many models (decision trees in this case) and combine their predictions. Such an approach is more reliable than using a single model. RF generates many uncorrelated decision trees by bootstrap aggregation, or “bagging” (randomly selecting samples from training data with replacement) and feature randomness (selecting a random subset of predictors for each tree). Predictions from all trees are then averaged for regression problems such as the one considered here. In contrast, XGboost uses the “boosting” concept, where trees are added sequentially, with each new tree “focusing” on the errors (residuals) of the previous trees.

We implemented RF (2000 trees), optimizing its parameters by RMSE using repeated cross-validation (threefold, 100 repeats) on the randomly-selected training half of the data. Performance (R^2^ and RMSE) was measured on the testing half of the data. This approach was designed to minimize the probability of “overfitting”. Robustness of RF predictions and performance metrics was assessed by applying the algorithm (with previously optimized parameters) to 300 random training/testing splits of the original data set. A similar approach of repeated cross-validation on training data was used to optimize the parameters of XGboost.

The MN/cell probability distribution metrics used as predictors (Table 1) are likely to be more accurate when the number of cells in a blood sample is large, whereas stronger random fluctuations are expected when the number of cells is small. To evaluate the effects of this phenomenon on neutron dose reconstruction accuracy, we repeated the machine learning and regression analyses on a subset of data that included 383 samples that contained ≥ 300 cells per sample. This cutoff excluded approximately 30% of the samples with the lowest numbers of cells per sample. The retained 383 samples had a median of 700 cells per sample, with a range of 308 to 3561. These large numbers represent sample sizes consistent with IAEA and ISO recommendations^38,39^.

## Results

**Figure 3:**
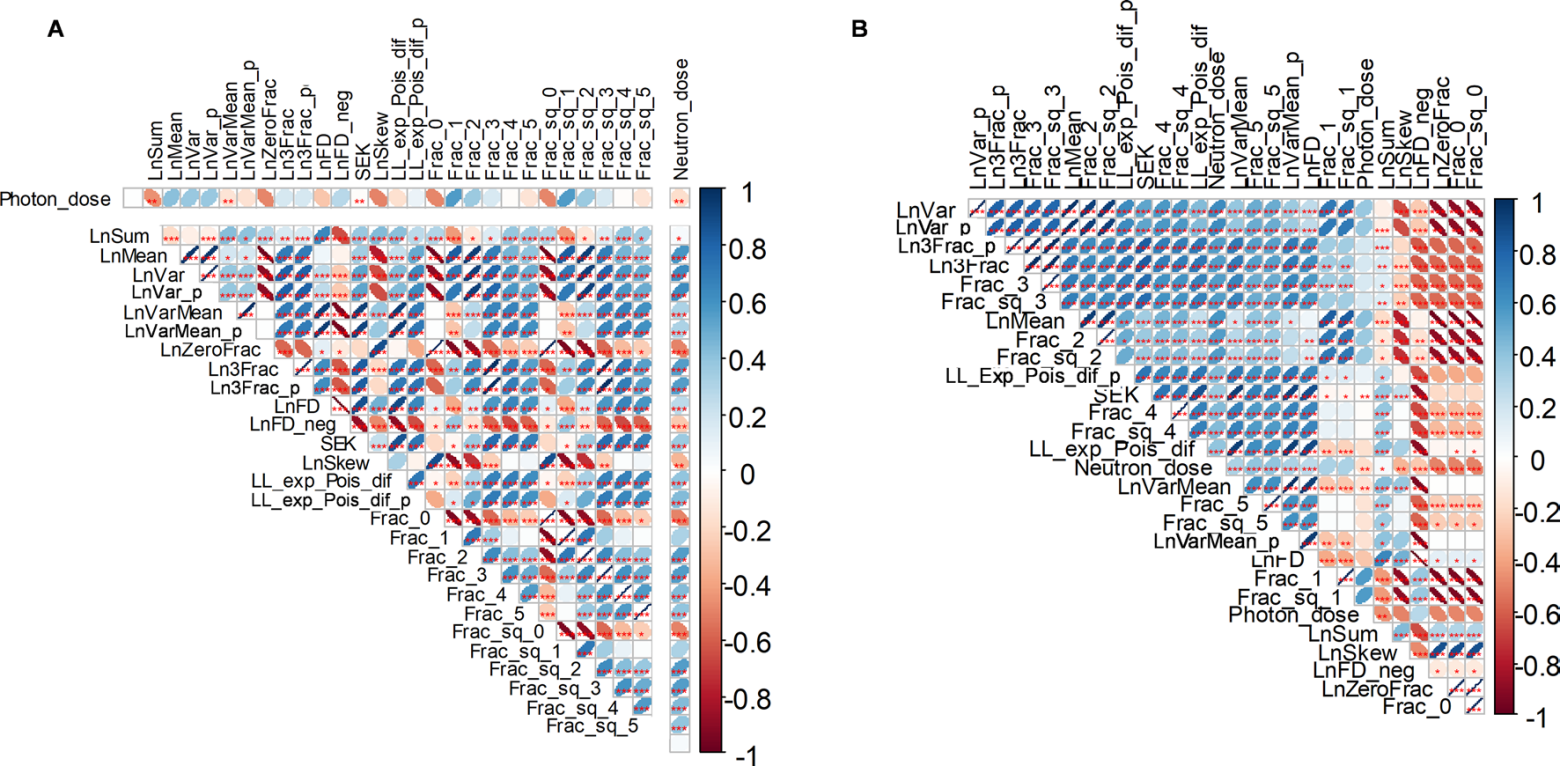
Matrix of Spearman’s correlation coefficients between all variables in the analyzed training data set. The meanings of all variables are provided in Table 1, and a color-coded correlation scale is provided on the right of the plot. Blue ellipses represent positive correlations, and red ones represent negative correlations. Darker color tones and narrower ellipses represent larger correlation coefficient magnitudes. Red star symbols indicate statistical significance levels: ***indicates p < 0.001, **indicates p < 0.01, *indicates p < 0.05, no stars indicate p > 0.05. These p-values here are intended only for visualization: since the correlations are pairwise, without correction for multiple testing, only 3-star significance levels are likely to indicate strong associations. Blank squares indicate correlation coefficients close to zero. (**A**) Default ordering of variables with photon and neutron doses in the top rows. (**B**) Ordering of variables by similar correlation coefficients to show variable groupings.

The correlation matrix of Spearman’s correlation coefficients between all variables in the training data set is shown in Fig. 3. For easier visualization, Spearman’s correlation coefficients for the photon dose and neutron dose with each variable on the training data set are also shown in Supplementary Table S3. Examination of these results, and implementation of the Boruta algorithm to rank predictors by their importance scores, narrowed the list of most useful predictors from 27 to 12. Listed in order of decreasing median importance over 100 Boruta repeats, these predictors and their median importance scores are as follows: LnVar 9.05, Ln3Frac 8.92, Ln3Frac_p 8.88, LL_exp_Pois_dif_p 8.68, LL_exp_Pois_dif 8.32, Frac_2 8.02, Frac_sq_2 7.96, Frac_0 7.28, Frac_3 6.82, Frac_sq_3 6.81, Frac_1 6.39, Frac_sq_1 6.33. Each of them is described in Table 1.

**Figure 4:**
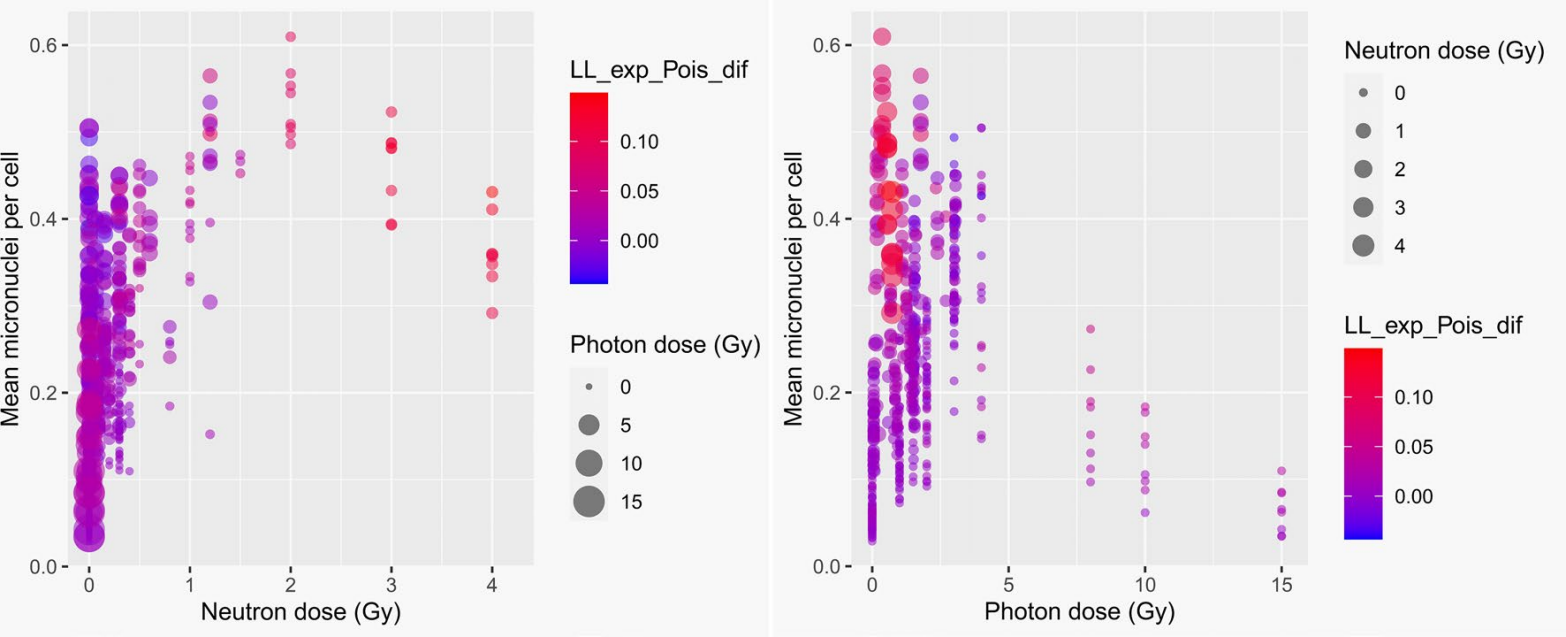
Visualization of how micronuclei per binucleated cell probability distribution shapes systematically differ after photon and neutrons exposures. Each circle represents a blood sample. The left and right panels show the same data set, but focus on different variables. LL_exp_Pois_dif (described in Table 1) is one of the predictors used in the machine learning analysis.

An example of how these predictors can be useful for neutron dose reconstruction in mixed neutron + photon exposures is shown graphically in Fig. 4 using the predictor LL_exp_Pois_dif. Figure 4 demonstrates that high values of this predictor (red circles) are associated mainly with those blood samples that were exposed to high neutron doses, regardless of how large the photon dose was. Therefore, when the observed probability distribution of MN/cell is much closer to Exponential than to Poisson, which is reflected in high values of LL_exp_Pois_dif, it is likely that a considerable dose of neutron radiation was involved.

Optimization of the random forest (RF) algorithm on training data using all retained predictors resulted in the following parameter values: number of variables to possibly split at in each node *mtry* = 12 (all predictors), splitting rule *splitrule* = variance, and minimal node size *min.node.size* = 1*.* The five most important predictors, listed in order of decreasing importance (assessed by permutation), were: LL_exp_Pois_dif_p, LnVar, LL_exp_Pois_dif, Ln3Frac_p, Ln3Frac.

**Figure 5:**
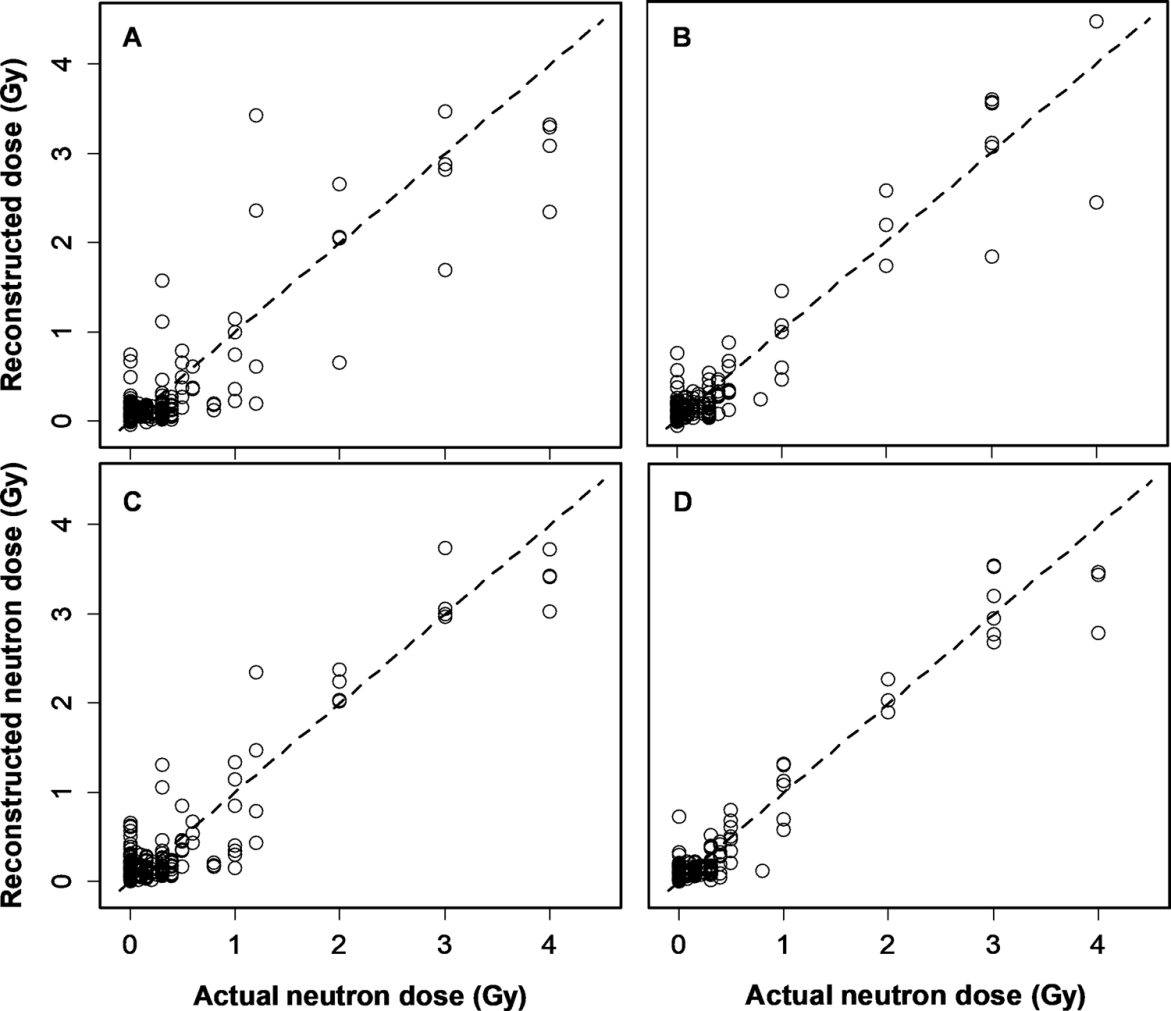
Neutron dose reconstruction results by the robust linear regression (RL) and random forest (RF) algorithms on testing data. (**A**) Results of analyzing the full data set by RL. R^2^ = 0.767, RMSE = 0.319 Gy. (**B**) Results of analyzing a subset of data where only blood samples with ≥ 300 cells were used by RL. R^2^ = 0.900, RMSE = 0.246 Gy. (**C**) Results of analyzing the full data set by RF. R^2^ = 0.860, RMSE = 0.248 Gy. (**D**) Results of analyzing a subset of data where only blood samples with ≥ 300 cells were used by RF. R^2^ = 0.936, RMSE = 0.189 Gy. In all panels, circles represent data points (blood samples), and the line represents theoretically perfect 1:1 correlation.

**Figure 6:**
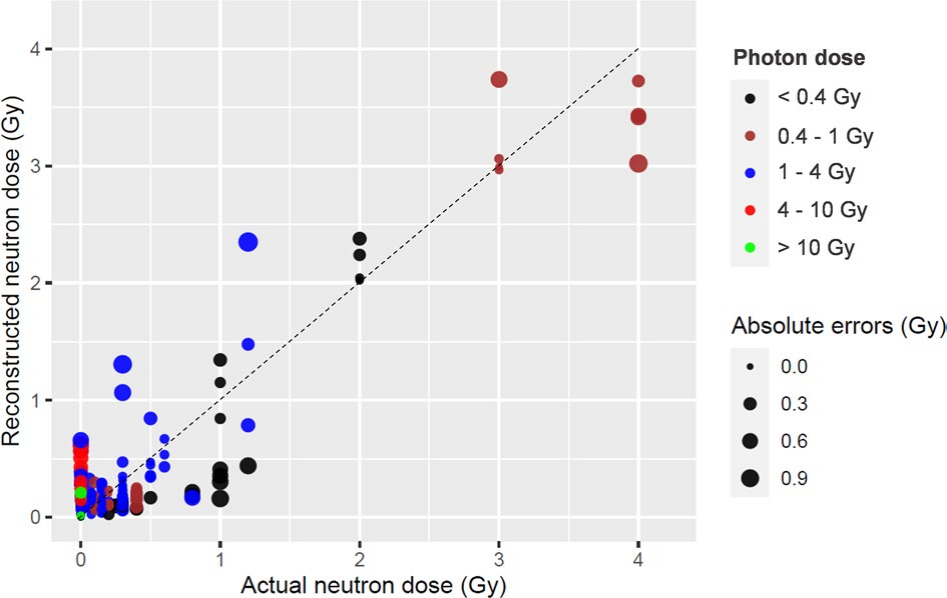
Visualization of the errors in neutron dose reconstruction by the random forest algorithm on testing data. Each circle represents a blood sample. These data points are the same as those in Fig. 5C, but absolute errors in neutron dose reconstruction are shown by circle size and the photon dose is shown by circle color (black: < 0.4 Gy, brown: 0.4–1 Gy, blue: 1–4 Gy, red: 4–10 Gy, green: > 10 Gy). The dashed line represents theoretically perfect 1:1 correlation.

The optimized RF achieved very good performance on the testing data, better than the performance of robust linear regression (Fig. 5). Linear regression of actual *vs*. RF reconstructed neutron doses on the testing data showed no significant systematic deviations: the intercept was − 0.0069 (standard error, SE = 0.017) which is close to the theoretically expected value of 0, and the slope was 0.971 (SE = 0.024) which is close to the expected value of 1. Therefore, neutron dose was reconstructed quite accurately, with neutrons being distinguished from pure photons even if the pure photon doses were very high (up to 15 Gy in some samples). This conclusion is supported by the finding that the Pearson correlation coefficient between photon dose and absolute error magnitude in neutron dose reconstruction was very small (− 0.0046). It is also supported by visualization of the errors in neutron dose reconstruction (Fig. 6), which shows that high doses of pure photons (red circles in the lower left corner of Fig. 6) are discriminated from high neutron doses.

Compared with RF, the optimized XGboost algorithm produced somewhat worse performance on the current data set: R^2^ on testing data was 0.832, and RMSE was 0.270 Gy. The finding that two different machine learning methods produced relatively similar accuracies on this data set suggests that details of the method’s assumptions are not crucial for neutron dose reconstruction. However, because RF performed better, we used this method rather than XGboost for further analyses.

RF performance was stable over 300 random training/testing splits of the data. Mean R^2^ on testing data over these repeats was 0.869 (standard deviation, SD = 0.028, range 0.761 to 0.919) and mean RMSE was 0.239 Gy (SD = 0.021, range 0.195 to 0.351). These findings suggest that the algorithm does not overfit the training data, is robust to small random fluctuations in the data composition, and has potential for application to other data sets.

RF analysis of a subset of data that included only those samples that contained ≥ 300 cells per sample resulted in even better performance than analysis of the full data set (Fig. 5). RF again outperformed robust linear regression on this subset of the data. Mean R^2^ for RF results on testing data over 300 random training/testing splits repeats was 0.928 (SD = 0.013, range 0.889, 0.958) and mean RMSE was 0.210 Gy (SD = 0.024, range 0.165, 0.292). The median of top 5 mean absolute error cases on testing data was reduced from 0.979 Gy on the full data set to 0.676 Gy on this subset of data. As for the analysis of the full data set, no significant systematic deviations between reconstructed and actual neutron doses were found, and there was no significant correlation between photon dose and absolute error magnitude in neutron dose reconstruction.

The improvements in RF performance on the subset of data with larger numbers of cells per sample, compared with the full data set, are not surprising because the predictor variables are affected by random data fluctuations, which are reduced at larger sample sizes. The set of predictors that passed the manual and Boruta selection steps was somewhat different for the analysis of the large sample subset, compared with the full data set. Listed in order of decreasing median importance over 100 Boruta repeats, these predictors and their median importance scores are as follows: LnVar 7.96, Ln3Frac_p 7.36, Ln3Frac 7.35, LL_exp_Pois_dif 7.31, LL_exp_Pois_dif_p 7.02, Frac_5 6.59, Frac_sq_5 6.58, Frac_sq_2 6.27, Frac_2 6.25, Frac_3 6.20, Frac_sq_3 6.19, Frac_4 5.90, Frac_sq_4 5.87, Frac_0 5.42, Frac_sq_1 5.08, Frac_1 5.05. Optimization of RF on training data using these predictors resulted in the following parameter values: number of variables to possibly split at in each node *mtry* = 8, splitting rule *splitrule* = extratrees, and minimal node size *min.node.size* = 1*.* The five most important predictors, listed in order of decreasing importance, were: LL_exp_Pois_dif, Ln3Frac, LL_exp_Pois_dif_p, Frac_5, Frac_sq_5.

## Discussion

The challenges posed by potential improvised nuclear device detonations or other types of malicious or accidental large-scale radiological events in populated areas drive the well-recognized need for high-throughput radiation biodosimetry^4–13^. Cytogenetic damage assays such as CBMN are good candidates for addressing these challenges because they are quite specific to radiation exposures, have strong dose responses, and are easy to automate^40^.

The traditional approach to radiation dose reconstruction based on cytogenetic damage involves scoring the damage in cells irradiated with various known doses, fitting a dose response function (e.g. linear quadratic) to these data, and using the fitted function (“calibration curve”) to “work backwards” to estimate the doses absorbed by test samples. This methodology, which is based on mean damage/cell yields, works well for estimating the total radiation dose. However, it runs into problems on complex tasks such as the one considered here—quantitative reconstruction of the neutron component in mixed neutron + photon exposures—because the mean damage yield does not provide any information about what type of radiation was involved.

Here we used a different strategy to address this problem. Its conceptual basis is the finding that MN/cell probability distributions from neutron exposures have different shapes (“tails”), compared with those from pure photon exposures (Fig. 2), even when mean MN yields are the same. These shapes of cytogenetic damage probability distributions provide a rich source of information, enabling different irradiation scenarios to be discriminated. For example, Figs. 2 and 4 show that MN/cell distribution shapes are quite different between radiation exposure situations where only photons are involved, *vs* those where a substantial component of neutrons is involved. After pure photon exposures, the distribution of MN/binucleated cells tends to be close to Poisson, whereas the presence of neutrons in the exposure shifts the distribution away from Poisson towards a distribution with larger tails, like Exponential.

To implement these concepts in practice, we used MN/cell probability distribution shape metrics (Table 1) as predictors for a machine learning algorithm, and radiation dose was the outcome (dependent variable). The algorithm was trained on data exposed to known radiation doses, and then used to estimate (predict) the doses absorbed by test samples. To our knowledge, we are the first team to implement this approach in radiation biodosimetry^17^. Here we built on this work by greatly expanding the photon and neutron dose ranges in the analyzed data set, and by refining the machine learning analysis methodologies for quantitative neutron dose reconstruction.

This strategy produced good accuracy of neutron dose component reconstruction in mixed neutron + photon exposures where the neutron contribution varied greatly (Figs. 5, 6). For example, mean MN/cell yields start to decrease at 3 Gy. The machine learning approach used here, which relies not only on the mean but also on other metrics, was able to partially compensate for this phenomenon and provide useful reconstructions up to 4 Gy.

In addition to these strengths, the study had certain limitations. Unfortunately, combinations of high neutron doses with high photon doses could not be tested due to severe damage to the cells, causing the majority of the cells to never reach the binucleated state, where micronuclei can be scored. Such combinations could be theoretically informative, but do not yield sufficient numbers of viable binucleated cells to be used for analysis. In addition, our data were generated by ex vivo blood irradiation, and more complexity would be expected for real exposures to mass radiological events. For example, high-dose exposures, particularly those with a high neutron contribution, will produce increased DNA damage and cell death in vivo and subsequent removal of cells from the circulating peripheral blood that could potentially lead to an under-estimation of the dose. Similarly, for the in vitro CBMN assay, highly damaged cells may fail to respond to mitogen stimulation and, therefore, fail to progress to the binucleated state and not be scored for MN yields. These processes reduce the MN assay’s accuracy at very high doses of neutrons and/or photons. Recently, we have addressed these issues by introducing caffeine into the culture^23^.

The RF algorithm used in this study also has inherent limitations, despite its popularity and widespread use. For example, RF cannot extrapolate beyond the range of the training data, can require more computational resources and time, and can be more difficult to interpret, compared with simpler models. On the current data, RF did not seem to discriminate well between 3 and 4 Gy of neutrons (Figs. 5, 6). However, an individual who received 3 or 4 Gy of neutrons (along with a likely photon component) will be so severely affected that medical decisions will not change in that case. We believe that what is really important is that even at the highest doses, the dose was not dramatically underestimated.

In summary, the main task here was to identify and quantify a neutron component in a mixed exposure using a novel machine learning approach to reconstruct the neutron dose. Its performance above 4 Gy of neutrons could not be tested using the current data set, and further experiments would be needed for such a high dose range. We also plan future experiments to investigate the potential effects of dose rate on the system. Overall, we believe that our results demonstrate the promising potential of machine learning to reconstruct the neutron dose component in clinically-relevant radiation exposure scenarios. Importantly, our approach discriminated well between neutron exposures and high doses of pure photons.

## Supplementary Information

Supplementary Dataset

Supplementary Tables
