# Supplementary material for: Machine learning methodology for high throughput personalized neutron dose reconstruction in mixed neutron + photon exposures
Source: Sci Rep. 2021 Feb 17;11:4022. doi: 10.1038/s41598-021-83575-5 (PMC7889851; doi:10.1038/s41598-021-83575-5)
Supplement: Supplementary file 2 — Supplementary Tables [file 41598_2021_83575_MOESM2_ESM.docx]

**Machine Learning Methodology for High Throughput Personalized Neutron Dose Reconstruction in Mixed Neutron+Photon Exposures**

**Supplementary Table 1.** **Summary of the analyzed data set on radiation-induced micronuclei in *ex vivo* irradiated peripheral blood lymphocytes.** The data for all blood samples with the same combination of neutron and photon doses were pooled (summed) to generate this summary table, but were analyzed separately. The full data set used for analysis is provided in the Supplementary_Dataset_File online. In some of the neutron+photon combinations, the photon dose was not measured directly, but was estimated based on the average photon/neutron ratio in the beam.

| **Photon dose (Gy)** | **Neutron dose (Gy)** | **Numbers of analyzed cells with the following number of micronuclei per cell:** | | | | | |
| --- | --- | --- | --- | --- | --- | --- | --- |
|  |  | **0** | **1** | **2** | **3** | **4** | **≥5** |
| 0.00 | 0.00 | 70018 | 3227 | 497 | 79 | 17 | 11 |
| 1.00 | 0.00 | 21023 | 2429 | 338 | 27 | 12 | 1 |
| 1.50 | 0.00 | 6871 | 1199 | 197 | 9 | 0 | 1 |
| 2.00 | 0.00 | 18236 | 3098 | 547 | 64 | 9 | 7 |
| 3.00 | 0.00 | 6732 | 2048 | 450 | 54 | 9 | 2 |
| 4.00 | 0.00 | 7316 | 1639 | 462 | 89 | 34 | 5 |
| 8.00 | 0.00 | 4155 | 354 | 140 | 45 | 14 | 5 |
| 10.0 | 0.00 | 4144 | 279 | 102 | 33 | 6 | 0 |
| 15.0 | 0.00 | 4310 | 141 | 40 | 13 | 8 | 4 |
| 3.01 | 0.06 | 1412 | 416 | 104 | 16 | 1 | 1 |
| 1.44 | 0.075 | 10365 | 1618 | 373 | 67 | 13 | 5 |
| 1.51 | 0.075 | 7787 | 1593 | 260 | 13 | 1 | 0 |
| 0.90 | 0.10 | 980 | 110 | 20 | 4 | 0 | 0 |
| 0.92 | 0.10 | 11468 | 1509 | 300 | 53 | 10 | 3 |
| 1.38 | 0.15 | 11164 | 1998 | 502 | 94 | 14 | 4 |
| 1.53 | 0.15 | 6897 | 1610 | 277 | 28 | 2 | 1 |
| 3.03 | 0.15 | 1363 | 439 | 98 | 14 | 2 | 0 |
| 0.036 | 0.20 | 785 | 79 | 16 | 2 | 0 | 0 |
| 0.80 | 0.20 | 882 | 123 | 19 | 3 | 1 | 0 |
| 0.84 | 0.20 | 10706 | 1607 | 378 | 82 | 9 | 3 |
| 1.80 | 0.20 | 361 | 79 | 11 | 4 | 1 | 0 |
| 0.046 | 0.25 | 6324 | 824 | 167 | 24 | 7 | 8 |
| 0.055 | 0.30 | 13712 | 1954 | 340 | 23 | 0 | 1 |
| 1.25 | 0.30 | 9746 | 1861 | 616 | 152 | 35 | 6 |
| 1.55 | 0.30 | 7136 | 1668 | 363 | 30 | 1 | 1 |
| 2.33 | 0.30 | 141 | 31 | 12 | 7 | 2 | 0 |
| 2.69 | 0.30 | 201 | 43 | 18 | 2 | 3 | 0 |
| 3.05 | 0.30 | 948 | 319 | 94 | 17 | 4 | 0 |
| 0.073 | 0.40 | 612 | 99 | 10 | 0 | 0 | 0 |
| 0.59 | 0.40 | 727 | 137 | 27 | 1 | 1 | 0 |
| 0.67 | 0.40 | 9433 | 1794 | 546 | 132 | 24 | 7 |
| 1.59 | 0.40 | 418 | 102 | 32 | 5 | 4 | 0 |
| 0.09 | 0.50 | 5323 | 981 | 226 | 47 | 32 | 21 |
| 1.09 | 0.50 | 6869 | 1682 | 666 | 152 | 43 | 14 |
| 2.39 | 0.60 | 1066 | 337 | 80 | 26 | 6 | 0 |
| 0.15 | 0.80 | 537 | 107 | 17 | 6 | 0 | 0 |
| 1.19 | 0.80 | 315 | 63 | 18 | 2 | 0 | 0 |
| 0.18 | 1.00 | 13722 | 3305 | 1361 | 422 | 84 | 40 |
| 0.22 | 1.20 | 381 | 100 | 30 | 10 | 0 | 2 |
| 1.78 | 1.20 | 1151 | 373 | 123 | 37 | 16 | 4 |
| 0.27 | 1.50 | 2248 | 658 | 258 | 65 | 22 | 11 |
| 0.36 | 2.00 | 8235 | 1597 | 1095 | 482 | 166 | 65 |
| 0.55 | 3.00 | 7209 | 899 | 660 | 369 | 163 | 79 |
| 0.73 | 4.00 | 5640 | 523 | 314 | 198 | 101 | 78 |

**Supplementary Table 2.** **Numbers of experimental data points corresponding to each combination of neutron dose and photon dose.** Blank cells in the table indicate no experiments with a given dose combination.

|  | **Neutron dose (Gy)** | | | | | | | | | | | | | | | | | |
| --- | --- | --- | --- | --- | --- | --- | --- | --- | --- | --- | --- | --- | --- | --- | --- | --- | --- | --- |
| **Photon dose (Gy)** | **0.00** | **0.06** | **0.08** | **0.10** | **0.15** | **0.20** | **0.25** | **0.3** | **0.4** | **0.5** | **0.6** | **0.8** | **1.0** | **1.2** | **1.5** | **2** | **3** | **4** |
| **0.00** | 65 |  |  |  |  |  |  |  |  |  |  |  |  |  |  |  |  |  |
| **0.04** |  |  |  |  |  | 3 |  |  |  |  |  |  |  |  |  |  |  |  |
| **0.05** |  |  |  |  |  |  | 3 |  |  |  |  |  |  |  |  |  |  |  |
| **0.05** |  |  |  |  |  |  |  | 31 |  |  |  |  |  |  |  |  |  |  |
| **0.07** |  |  |  |  |  |  |  |  | 3 |  |  |  |  |  |  |  |  |  |
| **0.09** |  |  |  |  |  |  |  |  |  | 3 |  |  |  |  |  |  |  |  |
| **0.15** |  |  |  |  |  |  |  |  |  |  |  | 3 |  |  |  |  |  |  |
| **0.18** |  |  |  |  |  |  |  |  |  |  |  |  | 11 |  |  |  |  |  |
| **0.22** |  |  |  |  |  |  |  |  |  |  |  |  |  | 3 |  |  |  |  |
| **0.27** |  |  |  |  |  |  |  |  |  |  |  |  |  |  | 3 |  |  |  |
| **0.36** |  |  |  |  |  |  |  |  |  |  |  |  |  |  |  | 8 |  |  |
| **0.55** |  |  |  |  |  |  |  |  |  |  |  |  |  |  |  |  | 8 |  |
| **0.59** |  |  |  |  |  |  |  |  | 3 |  |  |  |  |  |  |  |  |  |
| **0.67** |  |  |  |  |  |  |  |  | 12 |  |  |  |  |  |  |  |  |  |
| **0.73** |  |  |  |  |  |  |  |  |  |  |  |  |  |  |  |  |  | 8 |
| **0.80** |  |  |  |  |  | 3 |  |  |  |  |  |  |  |  |  |  |  |  |
| **0.84** |  |  |  |  |  | 12 |  |  |  |  |  |  |  |  |  |  |  |  |
| **0.90** |  |  |  | 3 |  |  |  |  |  |  |  |  |  |  |  |  |  |  |
| **0.92** |  |  |  | 12 |  |  |  |  |  |  |  |  |  |  |  |  |  |  |
| **1.00** | 37 |  |  |  |  |  |  |  |  |  |  |  |  |  |  |  |  |  |
| **1.09** |  |  |  |  |  |  |  |  |  | 12 |  |  |  |  |  |  |  |  |
| **1.19** |  |  |  |  |  |  |  |  |  |  |  | 2 |  |  |  |  |  |  |
| **1.25** |  |  |  |  |  |  |  | 12 |  |  |  |  |  |  |  |  |  |  |
| **1.38** |  |  |  |  | 12 |  |  |  |  |  |  |  |  |  |  |  |  |  |
| **1.44** |  |  | 12 |  |  |  |  |  |  |  |  |  |  |  |  |  |  |  |
| **1.50** | 18 |  |  |  |  |  |  |  |  |  |  |  |  |  |  |  |  |  |
| **1.51** |  |  | 22 |  |  |  |  |  |  |  |  |  |  |  |  |  |  |  |
| **1.53** |  |  |  |  | 22 |  |  |  |  |  |  |  |  |  |  |  |  |  |
| **1.55** |  |  |  |  |  |  |  | 22 |  |  |  |  |  |  |  |  |  |  |
| **1.59** |  |  |  |  |  |  |  |  | 3 |  |  |  |  |  |  |  |  |  |
| **1.78** |  |  |  |  |  |  |  |  |  |  |  |  |  | 9 |  |  |  |  |
| **1.80** |  |  |  |  |  | 3 |  |  |  |  |  |  |  |  |  |  |  |  |
| **2.00** | 45 |  |  |  |  |  |  |  |  |  |  |  |  |  |  |  |  |  |
| **2.33** |  |  |  |  |  |  |  | 1 |  |  |  |  |  |  |  |  |  |  |
| **2.39** |  |  |  |  |  |  |  |  |  |  | 6 |  |  |  |  |  |  |  |
| **2.69** |  |  |  |  |  |  |  | 2 |  |  |  |  |  |  |  |  |  |  |
| **3.00** | 34 |  |  |  |  |  |  |  |  |  |  |  |  |  |  |  |  |  |
| **3.01** |  | 9 |  |  |  |  |  |  |  |  |  |  |  |  |  |  |  |  |
| **3.03** |  |  |  |  | 9 |  |  |  |  |  |  |  |  |  |  |  |  |  |
| **3.05** |  |  |  |  |  |  |  | 9 |  |  |  |  |  |  |  |  |  |  |
| **4.00** | 2 |  |  |  |  |  |  |  |  |  |  |  |  |  |  |  |  |  |
| **8.00** | 8 |  |  |  |  |  |  |  |  |  |  |  |  |  |  |  |  |  |
| **10.00** | 8 |  |  |  |  |  |  |  |  |  |  |  |  |  |  |  |  |  |
| **15.00** | 8 |  |  |  |  |  |  |  |  |  |  |  |  |  |  |  |  |  |

**Supplementary Table 3. Spearman’s correlation coefficients for the photon dose and neutron dose with each variable on the training data set.**

| **Variable** | **Photon_dose** | **Neutron_dose** |
| --- | --- | --- |
| LnSum | -0.45 | 0.04 |
| LnMean | 0.41 | 0.59 |
| LnVar | 0.37 | 0.63 |
| LnVarMean | -0.15 | 0.32 |
| LnZeroFrac | -0.48 | -0.50 |
| Ln3Frac | 0.17 | 0.55 |
| LnFD | -0.28 | 0.26 |
| SEK | -0.02 | 0.37 |
| LnSkew | -0.49 | -0.34 |
| LL_exp_Pois_dif | -0.16 | 0.39 |
| LnVarMean_p | -0.15 | 0.31 |
| LL_exp_Pois_dif_p | 0.08 | 0.43 |
| Ln3Frac_p | 0.17 | 0.55 |
| LnFD_p | 0.28 | -0.26 |
| LnVar_p | 0.37 | 0.63 |
| Frac_0 | -0.48 | -0.50 |
| Frac_1 | 0.57 | 0.32 |
| Frac_2 | 0.37 | 0.56 |
| Frac_3 | 0.16 | 0.55 |
| Frac_4 | -0.02 | 0.44 |
| Frac_5 | -0.14 | 0.39 |
| Frac_sq_0 | -0.48 | -0.50 |
| Frac_sq_1 | 0.57 | 0.32 |
| Frac_sq_2 | 0.37 | 0.56 |
| Frac_sq_3 | 0.16 | 0.55 |
| Frac_sq_4 | -0.02 | 0.44 |
| Frac_sq_5 | -0.14 | 0.39 |
